# Planar Black Holes and Entanglement Entropy in Analog Gravity Models

**DOI:** 10.3390/e28030345

**Published:** 2026-03-19

**Authors:** Neven Bilic, Tobias Zingg

**Affiliations:** 1 Division of Theoretical Physics, Rudjer Bošković Institute, 10002 Zagreb, Croatia; 2 Department of Physics and Helsinki Institute of Physics, University of Helsinki, P.O. Box 64, FIN-00014 Helsinki, Finland

**Keywords:** analog gravity, planar black hole, holographic entanglement entropy

## Abstract

Via constructing an explicit Lagrangian for which the perturbation equations are analogs of a scalar field propagating in a planar black-hole space–time, it is found that all planar black holes conformal to a Painlevé–Gullstrand-type line element can be realized as analog metrics. We also introduce the concept of holographic entanglement entropy for planar black-hole space–times. This is valid for an arbitrary choice of conformal and blackening factor, thereby vastly extending the number of known examples of explicitly known analog metrics.

## 1. Introduction

For certain condensed matter systems, the Lagrangians describing them have a property that small perturbations around a given background are described by the equations of motion of a field propagating in curved space–time. Thus, these systems may serve as ‘analogs’ of phenomena in gravitational physics and could, in principle, be employed to simulate gravity in tabletop experiments. Though already known theoretically since the 1980s [1,2], it is only in recent years that this approach to simulating gravity has attracted more attention, mostly due to new technologies—in particular, in dealing with Bose–Einstein condensates (BECs) or cold atom systems—having been developed and making these kinds of experiments more accessible [3,4,5,6,7,8,9].

However, it should be noted that, a priori, not all interesting geometries can be mimicked by analog geometries. For example, counting the available degrees of freedom, in general relativity (GR) and 3+1 dimensions, we have four degrees of freedom per point in space–time: ten independent components of a general metric minus six owing to the six independent Einstein equations. Whereas an analog metric basically depends on two independent functions, the scalar potential θ that generates the flow velocities and the speed of sound *c*. Thus, the basic analog gravity setup involving a single scalar field, which is also what we will consider in the following, can not reproduce all possible metrics that could be derived from GR. However, as additional degrees of freedom enter by coupling to an external potential, which is assumed to be freely tunable, the setup considered is actually more than sufficient to mimic the most important phenomena, such as black holes, FRW cosmology, and even some aspects of semiclassical quantum gravity, such as Hawking radiation.

As these phenomena are all of central importance in gravity physics, it is desirable to extend the class of analog gravity systems to as many metrics as possible. Besides astronomic observations, analog gravity provides the only way to experimentally test such predictions in a lab environment. In this paper, we follow the formalism developed in [10] where it was restricted to a planar AdS_5_ black hole (BH), and extend the applicability of analog gravity by demonstrating that it can potentially capture all the phenomena described by a field propagating in any space–time that is conformal to a rather generic stationary planar BH, for an arbitrary choice of blackening factor. Our paper provides a generalization and adds to the examples of planar space–times that have already been found to have an analog dual [11,12,13].

We aim to generalize possible analog planar geometric structures that could, in principle, be accomplished by a suitable design of the fluid flow. In analog gravity, the planar BHs may appear if a fluid flows along one coordinate dimension so that two other space dimensions become irrelevant. The reasons for considering primarily planar black holes are threefold.

First, black holes in fewer than three dimensions have been extensively studied in the literature (see, e.g., [14,15]), although the observed astrophysical black holes are three-dimensional. Besides, geometric structures in the form of a planar BH may have interesting applications in condensed matter physics (see, e.g., [16]), in particular in the 2+1-dimensional superconductor [17,18,19].

Second, the effects of the curvature of the horizon, e.g., spherical or hyperbolic, are secondary in most situations of practical interest and could, effectively, at any rate be absorbed into a redefinition of the effective mass of the scalar perturbation. Furthermore, experiments that simulate horizon-related phenomena, such as the Hawking effect, in analog systems involving the flow of water in a basin, Bose–Einstein condensates, or cold atoms in a trap often employ a linear setup, making planar black holes a more suitable choice for practical purposes [20,21]. In ultrarelativistic heavy-ion collisions, the fluid of particles is predominantly produced along one space dimension. Hence, the effective space–time is 1+1, which is equivalent to planar geometry where two space dimensions may be ignored.

Third, we provide a proof of concept on how analog Lagrangians for a general class of space–times, not just with individual metrics, can be constructed, thereby significantly extending the menagerie of known analog black-hole metrics. The case of generic black-hole space–times, not only for specific blackening factors, provides a suitable starting point due to its relevance for phenomena involving an event horizon, which is one of the main research points in analog gravity experiments, and due to the previous work on which to build.

The paper is organized as follows. In Section 2 we define the geometry and its conformally rescaled metric. In Section 3 we outline a field-theory description of a fluid and derive the propagation equation for acoustic perturbations. The main result follows in Section 4, where we show how a generic planar black-hole metric can be mapped to the effective geometry of a fluid in which acoustic perturbations propagate. In Section 5 we define the analog entanglement entropy for a general analog planar BH metric and compute it numerically for an analog planar AdS_5_ BH. Concluding remarks are given in Section 6.

We adopt a convention in which the speed of light and Planck constant *ℏ* are set to unity, where *c* denotes the speed of sound and the metric signature is ‘mostly plus’, i.e., {−,+,…,+}.

## 2. Conformal Rescaling

For the purpose of being self-contained, we summarize a result from [20], which shows how an additional degree of freedom in the form of a conformal factor can be introduced into an analog metric.

Consider a space–time in n+1 dimensions conformal to a rather generic stationary planar BH metric, which, for later convenience, we take to be parameterized as

(1) $$\begin{array}{c} ds^{2}=G_{\mu \nu }dx^{\mu }dx^{\nu }=\frac{\Omega {\left(t,x,y,z\right)}^{2}}{\sqrt{1-\gamma (z)}}\left[-\gamma \left(z\right)dt^{2}+\frac{dz^{2}}{\gamma (z)}+dx^{2}\right], \end{array}$$

where the function γ is referred to as the ‘blackening factor’. The metric as written in (1) refers to a general planar metric. In the next section, we will show how this form of metric can be achieved as an effective acoustic metric with the help of a specifically designed fluid flow. If there is a horizon located at z=ℓ, where γ(ℓ)=0, the outside region is characterized by γ>0. A canonical scalar field φ propagating in this background with effective mass $m_{\mathrm{eff}}$ satisfies the equation of motion

(2) $$\begin{array}{c} □\varphi -m_{\mathrm{eff}}^{2}\varphi \equiv \frac{1}{\sqrt{\left|G\right|}}\partial_{\mu }\left(\sqrt{\left|G\right|}\;G^{\mu \nu }\partial_{\nu }\varphi \right)-m_{\mathrm{eff}}^{2}\varphi =0, \end{array}$$

where it is assumed that $m_{\mathrm{eff}}$ and φ depend on the coordinates *t*, *x*, *y*, and *z*. The symbol □ denotes the Klein–Gordon operator in curved space with the metrics $G_{\mu \nu }$. Via the rescaling (note that we use a slightly different convention than in [20]) $\varphi =\Omega^{\frac{1-n}{2}}\tilde{\varphi }$, this equation is equivalent to the conformally rescaled equation of motion [20,22]

(3) $$\begin{array}{c} \tilde{□}\tilde{\varphi }-{\tilde{m}}_{\mathrm{eff}}^{2}\tilde{\varphi }=0, \end{array}$$

where it is assumed that ${\tilde{m}}_{\mathrm{eff}}$ and $\tilde{\varphi }$ depend on *t*, *x*, *y*, and *z*. The symbol $\tilde{□}$ denotes the Klein–Gordon operator in curved space with the metrics ${\tilde{G}}_{\mu \nu }=\Omega^{-2}G_{\mu \nu }$. The rescaled field $\tilde{\varphi }$ is propagating in the background geometry with a conformally rescaled line element

(4) $$\begin{array}{c} d{\tilde{s}}^{2}=\Omega {\left(t,x,y,z\right)}^{-2}ds^{2}={\tilde{G}}_{\mu \nu }dx^{\mu }dx^{\nu }=\frac{1}{\sqrt{1-\gamma (z)}}\left[-\gamma \left(z\right)dt^{2}+\frac{dz^{2}}{\gamma (z)}+dx^{2}\right], \end{array}$$

and effective mass squared

(5) $$\begin{array}{c} {\tilde{m}}_{\mathrm{eff}}^{2}=\Omega^{2}m_{\mathrm{eff}}^{2}+\Omega^{1/2-n/2}\tilde{□}\Omega^{n/2-1/2}. \end{array}$$

## 3. The Lagrangian

We begin this section by introducing the Lagrangian formalism suitable for description of generally nonisentropic fluids. We mainly use notation and definitions from previous work (see, e.g., [10,23,24]), which are standard for this system. Consider a Lagrangian as follows:

(6) $$\begin{array}{c} L=F(\chi )-V(\theta ,t,x,y,z), \end{array}$$

where θ is a dimensionless scalar field. The quantity *F* is an arbitrary function of the kinetic energy term

(7) $$\begin{array}{c} \chi =-g^{\mu \nu }\theta_{,\mu }\theta_{,\nu }, \end{array}$$

where $g^{\mu \nu }$ is the inverse metric of the background space–time. We will shortly demonstrate that the Lagrangian (6) is associated with any perfect fluid given its equation of state. Besides, it has been shown that this Lagrangian, with the kinetic term *F* only, in the so-called Thomas–Fermi approximation corresponds to a canonical complex field Lagrangian that describes a Bose–Einstein condensate (see, e.g., [25,26]).

The energy–momentum tensor for (6) is

(8) $$\begin{array}{c} T_{\mu \nu }=2L_{\chi }\theta_{,\mu }\theta_{,\nu }+Lg_{\mu \nu }, \end{array}$$

where the subscript χ denotes a partial derivative with respect to χ. For χ>0, this energy–momentum tensor will describe a perfect fluid if we identify the pressure and energy density as

(9) $$\begin{array}{c} p=L, \end{array}$$

(10) $$\begin{array}{c} \rho =2\chi L_{\chi }-L, \end{array}$$

and the fluid velocity vector as

(11) $$\begin{array}{c} u_{\mu }=\frac{\theta_{,\mu }}{\sqrt{\chi }}. \end{array}$$

This equation describes the so-called ‘potential flow’. Solutions of this form are the relativistic analog of potential flow in non-relativistic fluid dynamics [27] and are usually ascribed to isentropic and irrotational flows. Isentropic flow is characterized by the vanishing of the gradient $s_{,\mu }=0$, with *s* being the specific entropy, i.e., the entropy per particle. In general, a flow may be non-isentropic and have a non-vanishing vorticity $\omega_{\mu \nu }$ defined as

(12) $$\omega_{\mu \nu }=h_{\mu }^{\rho }h_{\nu }^{\sigma }u_{\left[\rho ;\sigma \right]},$$

where

(13) $$h_{\nu }^{\mu }=\delta_{\nu }^{\mu }+u^{\mu }u_{\nu }.$$

This tensor projects an arbitrary vector in space–time into its component in the subspace orthogonal to $u^{\mu }$. If the conditions of isentropy and vanishing vorticity are assumed, the velocity field may be expressed by

(14) $$wu_{\mu }=\theta_{,\mu }$$

where θ is the velocity potential and *w* is the specific enthalpy. The reverse of the above statement is not true: a potential flow alone implies only vanishing vorticity and implies neither isentropy nor particle number conservation. In a potential flow, as may be easily shown [10], the entropy gradient is proportional to the gradient of the potential, i.e.,

(15) $$s_{,\mu }=w^{-1}u^{\nu }s_{,\nu }\theta_{,\mu }.$$

The assumption in (14) is equivalent to (11) if we identify

(16) $$\begin{array}{c} w\equiv \frac{p+\rho }{n}=\sqrt{\chi }. \end{array}$$

Hence, the potential flow is automatically satisfied in the field-theory formalism with a scalar field θ playing the role of the velocity potential. Furthermore, in view of (16) with (9) and (10), we identify the particle number density as

(17) $$\begin{array}{c} n=2\sqrt{\chi }L_{\chi }. \end{array}$$

This is consistent with the Gibbs relation

(18) $$\begin{array}{c} dp=ndw-nTds=L_{\chi }d\chi +L_{\theta }d\theta , \end{array}$$

when a functional relationship s=s(θ) is assumed.

Thus, we have constructed a field-theory description of a fluid. Following [10], the ideal irrotational fluid will satisfy the Euler equation—i.e., the energy momentum conservation—if, in addition to the potential flow in Equation (11), the field satisfies the equation of motion

(19) $${\left(2L_{\chi }g^{\mu \nu }\theta_{,\nu }\right)}_{;\mu }+\frac{\partial L}{\partial \theta }=0.$$

Using (11) and (17), this equation can be written as

(20) $$\begin{array}{ccc} {\left(nu^{\mu }\right)}_{;\mu } & = & \frac{\partial V}{\partial \theta }. \end{array}$$

Next, we briefly describe the derivation of the propagation equation for linear perturbations of a nonisentropic flow assuming a fixed background geometry. Given some average bulk motion represented by *p*, *n*, and $u^{\mu }$, following the standard procedure [28] we make a replacement

(21) $$p\to p+\delta p,\;n\to n+\delta n,\;u^{\mu }\to u^{\mu }+\delta u^{\mu },$$

where the perturbations δp, δn, and $\delta u^{\mu }$ are induced by a small perturbation $\theta =\theta_{0}+\delta \theta$, around the background $\theta_{0}$. From (14) we find

(22) $$\delta w=-u^{\mu }\delta \theta_{,\mu },$$

(23) $$w\delta u^{\mu }=\left(g^{\mu \nu }+u^{\mu }u^{\nu }\right)\delta \theta_{,\nu }.$$

Using this and (21) Equation (20) at linear order yields

(24) $${\left(f^{\mu \nu }\delta \theta_{,\nu }\right)}_{;\mu }+\left[{\left(\frac{\partial n}{\partial \theta }u^{\mu }\right)}_{;\mu }-\left(\frac{\partial^{2}V}{\partial \theta^{2}}\right)\right]\delta \theta =0,$$

where

(25) $$f^{\mu \nu }=\frac{n}{w}\left[g^{\mu \nu }+\left(1-\frac{w}{n}\frac{\partial n}{\partial w}\right)u^{\mu }u^{\nu }\right].$$

Then, it may be easily shown that Equation (24) can be recast into the form

(26) $$\begin{array}{c} |\tilde{G}{|}^{-1/2}(|\tilde{G}{{|}^{1/2}{\tilde{G}}^{\mu \nu }\delta \theta_{,\nu })}_{;\mu }-m_{\mathrm{eff}}^{2}\delta \theta =0, \end{array}$$

where ${\tilde{G}}^{\mu \nu }$ is the inverse of the relativistic acoustic metric [23]

(27) $$\begin{array}{c} {\tilde{G}}_{\mu \nu }=\frac{n}{m^{2}wc}\left[g_{\mu \nu }+\left(1-c^{2}\right)u_{\mu }u_{\nu }\right] \end{array}$$

with determinant $\tilde{G}$. Hence, the acoustic perturbation δθ is a scalar field equivalent to the field $\tilde{\varphi }$ in Section 2, that satisfies the Klein–Gordon Equation (3) equivalent to (26). Note that in this section, unlike in the Section 2, the metric ${\tilde{G}}_{\mu \nu }$ with capital *G* denotes the effective acoustic metric, whereas the metric of the background space–time is denoted by $g_{\mu \nu }$. The quantity ${\tilde{m}}_{\mathrm{eff}}$ in Equation (26) is henceforth assumed to depend on *t*, *x*, *y*, and *z*. An arbitrary mass parameter *m* in (27) is introduced to make the metric in ${\tilde{G}}_{\mu \nu }$ dimensionless, and *c* is the speed of sound defined by

(28) $$\begin{array}{c} {\left.c^{2}\equiv \frac{\partial p}{\partial \rho }\right|}_{s}={\left.\frac{n}{w}\frac{\partial w}{\partial n}\right|}_{s}. \end{array}$$

From now on, we will assume that the sound speed satisfies

(29) 0≤c≤1.

The quantity $m_{\mathrm{eff}}$ is the effective mass defined by

(30) $$\begin{array}{c} m^{2}\sqrt{|\tilde{G}|}\;m_{\mathrm{eff}}^{2}={\left.\frac{\partial^{2}V}{\partial \theta^{2}}\right|}_{\theta_{0}}. \end{array}$$

Using (6)–(11), one can derive the relation

(31) $$\begin{array}{c} m^{2}\sqrt{|\tilde{G}|}\;{\tilde{G}}^{\mu \nu }={\left.-\frac{\partial^{2}F}{\partial \theta_{,\nu }\partial \theta_{,\mu }}\right|}_{\theta_{0}}, \end{array}$$

also derived by Babichev et al. [24] in a different context.

Here, it is worth mentioning the diffeomorphism invariance of our analog model. The Lagrangian L defined in (6) with (7) is a scalar, so the action $S=\int d^{4}x\sqrt{-g}L$ and the corresponding field equations are invariant under general coordinate transformation. The perfect fluid stress tensor $T_{\mu \nu }$, defined above with scalar variables ρ and *p* and the four-vector $u^{\mu }$, is a covariant tensor. Furthermore, the manipulations leading to the acoustic metric in (27) are fully covariant. Hence, the hydrodynamic model that stems from the on-shell Lagrangian and the derived acoustic geometry are 4D-diffeomorphism invariant.

## 4. Relativistic Acoustic Metric

Building on [10], we now proceed to show that an acoustic perturbation in a fluid—the dynamics of which is described by an explicit field-theory Lagrangian—can be realized as a scalar field propagating in the background (Equation (4)). This extends the procedure developed in [10], which was restricted to a planar AdS_5_ BH with

(32) $$\gamma \left(z\right)=1-\frac{z^{4}}{\ell^{4}},$$

where the function γ(z) is the blackening coefficient in the planar AdS_5_ metric with the horizon at z=ℓ, similar to the Schwarzschild metric where γ depends only on *r* with the horizon at $r=r_{\mathrm{Sch}}$. Here we will show that the formalism can be generalized to simulate metrics of the form in (4) with an arbitrary blackening factor γ(z) subject only to the restriction

(33) γ≤1.

In particular, we will show that an acoustic perturbation propagating in a fluid described by the Lagrangian of the form in (6) represents an analog dual of a scalar field propagating in the background in (4). In other words, if a fluid is described by the Lagrangian in (6), the dynamics of acoustic perturbations described by (26)–(30) will have the form of the Klein–Gordon equation (Equation (3)) in a curved space–time described by the line element in (4).

The first step is to bring the Metric (4) to a form that can be compared with the acoustic Metric (27). For this purpose, we make the following coordinate transformation from the coordinates *t* and *z* to new coordinates $\tilde{t}$ and $\tilde{z}$, keeping *x* and *y* intact,

(34) $$\begin{array}{c} t=\tilde{t}+f\left(z\right),\;z=g\left(\tilde{z}\right). \end{array}$$

Then, the line element from (4) takes the form

(35) $$\begin{array}{ccc} d{\tilde{s}}^{2} & = & \frac{1}{\sqrt{1-\gamma }}\{-d{\tilde{t}}^{2}+d{\tilde{z}}^{2}+dx^{2}\\ & & +\left[\left(1-\gamma \right)d{\tilde{t}}^{2}-2\sqrt{\left(1-\gamma \right)\left(c^{2}-\gamma \right)}d\tilde{t}d\tilde{z}+\left(c^{2}-\gamma \right)d{\tilde{z}}^{2}\right]\}, \end{array}$$

where

(36) $$\begin{array}{c} \frac{dg}{d\tilde{z}}=c,\;\frac{df}{dz}=\frac{\sqrt{\left(1-\gamma \right)\left(c^{2}-\gamma \right)}}{c\gamma }. \end{array}$$

Comparing with (27) allows one to read off the non-vanishing components of the four-velocity,

(37) $$\begin{array}{c} u_{\tilde{t}}=\sqrt{\frac{1-\gamma }{1-c^{2}}},\;u_{\tilde{z}}=-\sqrt{\frac{c^{2}-\gamma }{1-c^{2}}}. \end{array}$$

These equations imply

(38) $$\gamma \leq c^{2}\leq 1.$$

Next, assuming the potential flow from (14), we derive closed expressions for *w*, *n*, and *c* in terms of the variable $\tilde{z}$. Since the metric is stationary, the velocity potential must be of the form

(39) $$\theta =m\tilde{t}+h\left(z\right),$$

where *m* is an arbitrary mass and h(z) is a function of $\tilde{z}$ through $z=g(\tilde{z})$. The specific enthalpy is then given by

(40) $$\begin{array}{c} w=\frac{m}{u_{\tilde{t}}}=m\sqrt{\frac{1-c^{2}}{1-\gamma }} \end{array}$$

and the function h(z) is determined through

(41) $$\begin{array}{c} \frac{dh}{dz}=-\frac{m}{c}\sqrt{\frac{c^{2}-\gamma }{1-\gamma }}. \end{array}$$

From the definition (28) it follows

(42) $$\begin{array}{c} c^{2}=\frac{n}{w}\frac{\partial_{\tilde{z}}w}{\partial_{\tilde{z}}n}. \end{array}$$

This implies that the sound speed *c* must satisfy the differential equation

(43) $$\begin{array}{c} \frac{\partial }{\partial \tilde{z}}c^{2}=\left(c^{2}-\frac{1}{2}\right)\frac{\partial }{\partial \tilde{z}}\ln\left(1-\gamma \right), \end{array}$$

with the solution

(44) $$\begin{array}{c} c^{2}=c_{1}\left(1-\gamma \right)+\frac{1}{2}. \end{array}$$

Due to the requirement in (29), the integration constant $c_{1}$ is restricted to

(45) $$-\frac{1}{2(1-\gamma_{\min})}\leq c_{1}\leq \frac{1}{2(1-\gamma_{\min})},$$

where $\gamma_{\min}$ is the minimal value of γ. If we do not want to cover the region within the horizon, we can choose $\gamma_{\min}=0$, in which case we have $-1/2\leq c_{1}\leq 1/2$. Considering that the sound speed must satisfy both (38) and (44), it is unlikely that with a single choice of $c_{1}$ we could cover the whole physical range z>0. We will elaborate more on this below. Furthermore, in view of (27), (35) and (44), we can write the enthalpy and particle number density as

(46) $$\begin{array}{c} w=m\sqrt{\frac{1}{2(1-\gamma )}-c_{1}}, \end{array}$$

(47) $$\begin{array}{c} n=m^{3}\sqrt{\frac{1}{4{\left(1-\gamma \right)}^{2}}-c_{1}^{2}}=m^{2}w\sqrt{\frac{1}{2(1-\gamma )}+c_{1}}. \end{array}$$

In principle, $c_{1}$ could be a function of *s*. However, since *w* and *s* are considered as independent variables, the right-hand side of (46) admits no explicit *s*-dependence. Hence, a consistent choice is $c_{1}\equiv \mathrm{const}$. From (46) and (47) it follows that

(48) $$\begin{array}{c} n\frac{\partial w}{\partial \tilde{z}}=\frac{m^{2}}{2}\sqrt{\frac{1}{2(1-\gamma )}+c_{1}}\;\frac{\partial w^{2}}{\partial \tilde{z}}=\frac{m^{4}}{3}\frac{\partial }{\partial \tilde{z}}{\left(\frac{1}{2(1-\gamma )}+c_{1}\right)}^{3/2}. \end{array}$$

Then, according to (18) the pressure reads as

(49) $$\begin{array}{c} p=\frac{m^{4}}{3}{\left(\frac{1}{2(1-\gamma )}+c_{1}\right)}^{3/2}-c_{2}\left(s\right), \end{array}$$

where $c_{2}\left(s\right)$ is an arbitrary function of *s*. In view of (46) the pressure can also be expressed as

(50) $$\begin{array}{c} p=\frac{m^{4}}{3}{\left(\frac{w^{2}}{m^{2}}+2c_{1}\right)}^{3/2}-c_{2}\left(s\right). \end{array}$$

This expression is precisely of the form in (6) in which

(51) $$\begin{array}{c} F\left(\chi \right)=\frac{m^{4}}{3}{\left(\frac{\chi }{m^{2}}+2c_{1}\right)}^{3/2}, \end{array}$$

where $c_{2}$ is identified with *V* and the specific enthalpy with $\sqrt{\chi }$ as in (16).

Therefore, we have shown that the Lagrangian (Equation (6)) with (51) can be used to construct an analog model for a scalar field propagating in the Metric (4) with an arbitrary γ(z). However, as mentioned previously, with a specific choice of constant $c_{1}$, we would, in general, only cover a part of the range z≥0. If we require that the horizon γ=0 lies within the allowed range, we will find a constraint as to how close to the limit γ=1 our analog metric is applicable. Suppose we choose to cover only the outside region, so that $c_{1}$ is restricted to the interval [−1/2,1/2]. As a consequence of Equation (38), our analog model will break down at a point $z=z_{\min}$, which is the maximal root of the algebraic equation γ(z)=2/3. This equation follows from imposing that $c^{2}=\gamma$ and Equation (44) with maximal $c_{1}=1/2$. For example, for a planar AdS_5_ BH with $\gamma =1-z^{4}/\ell^{4}$, one finds $z_{\min}=\ell /3^{1/4}$. Similarly, if we chose to cover the entire region within the horizon up to z=∞, the algebraic equation would read as γ(z)=1/2. Then, for a planar AdS_5_, we would obtain $z_{\min}=\ell /2^{1/4}$.

It is worth noting that the Lagrangian (Equation (6)) with (51) has the same functional dependence on χ as the one found in [10], where it was derived from the requirement that the analog metric correctly reproduces the planar AdS_5_ BH. Hence, the functional form of (51) is generic. However, the fluid dynamics is not completely determined unless the potential V(θ) is specified because the flow velocity components are fully determined by the velocity potential θ, which solves the field equations. To find a solution to the field equations, we need to specify the potential V(θ) which will be done in the following section.

### The Potential

Recall that we are considering a scalar field $\theta =\theta_{0}+\delta \theta$, i.e., a small acoustic perturbation δθ around a fixed background $\theta_{0}$. The equation of motion of this perturbation is an analog model of a particle propagating in the curved space–time. Then, the potential *V* has to meet a requirement that its first derivative, when evaluated on the background in (39), is determined by Equation (20). In applications where one wishes to simulate a specific effective mass (such as, e.g., in [29]) in addition to a specific metric, Equation (30) requires imposing conditions on the second derivative of *V*. Thus, the potential *V* has to be chosen such that

(52) $$\begin{array}{c} {\left.\frac{\partial V}{\partial \theta }\right|}_{\theta =\theta_{0}}={\left(nu^{\mu }\right)}_{;\mu };,\;{\left.\frac{\partial^{2}V}{\partial \theta^{2}}\right|}_{\theta =\theta_{0}}=\sqrt{|\tilde{G}|}m^{2}m_{\mathrm{eff}}\left(\tilde{t},x,y,\tilde{z}\right). \end{array}$$

where the new coordinates $\tilde{t}$ and $\tilde{z}$ are defined by the coordinate transformation in (34). In principle, one could satisfy these conditions in many ways. Quite generally, a suitable potential can be written as

(53) $$\begin{array}{c} V=\alpha \left(\tilde{z}\right)\theta f_{1}\left(\theta /\theta_{0}\right)+\beta \left(\tilde{z}\right)\theta^{2}f_{2}\left(\theta /\theta_{0}\right) \end{array}$$

where $f_{1}\left(x\right)$ and $f_{2}\left(x\right)$ are arbitrary functions which at x=1 (i.e., when $\theta =\theta_{0}$) satisfy

(54) $$\begin{array}{c} {\left.{\left(xf_{1}\left(x\right)\right)}^{\prime \prime }\right|}_{x=1}=0\;,\;{\left.{\left(x^{2}f_{2}\left(x\right)\right)}^{\prime }\right|}_{x=1}=0\;. \end{array}$$

and $\alpha \left(\tilde{z}\right),\beta \left(\tilde{z}\right)$ are chosen to match (52). Therefore, the potential *V* will generally have to be chosen as coordinate-dependent. This would present no real obstacle from a practical point of view, as experimental setups for analog gravity with moving and oscillating horizons are already being conducted (see Refs. [5,6]), and time- and position-dependent external potentials could be simulated with the same setup.

From a theoretical point of view, there could be some caveat that limits the choice of potentials, thatcomes from the condition that the Gibbs relation (18) must hold. At first sight, it may seem a bit odd how the relation containing only two degrees of freedom could be satisfied with a generic potential V(θ,t,z,x). However, one has to keep in mind that the functional identities in Section 3 are independent of the specific coordinate dependence of the potential, and the crucial point is that the Gibbs relation (18) has to hold as an on-shell functional identity. This is to say that it must be possible to express the pressure *p* as a functional depending on two variables *w* and *s* which are defined on the function space of solutions to the equations of motion. This reduces the effective number of degrees of freedom. (Note that the the Gibbs relation need not hold for a generic field that does not satisfy the equations of motion.) In practice, however, it could be rather non-trivial to check (18) explicitly, and the following construction might be more convenient.

Assume a Lagrangian with no explicit coordinate dependence of the form

(55) $$\begin{array}{c} L=F(\chi )-V(\theta ). \end{array}$$

The Gibbs relation (18) is now automatically satisfied and for a solution $\theta_{0}$ of the equations of motion in (20), the analog metric and effective mass for a perturbation follow from (31) and (30). In this situation, one can proceed to construct a potential *V* that reproduces the desired analog metric in a way similar to [10], where it has been worked out for the case of a planar BH in AdS space–time. In order to then explicitly match the effective mass to a desired value, consider the Lagrangian (55) changed by an $O{\left(\theta -\theta_{0}\right)}^{2}$ deformation around the found background solution $\theta_{0}$, e.g.,

(56) $$\begin{array}{c} L^{\prime }=F\left(\chi \right)-V\left(\theta \right)-\frac{a(\theta ,t,x,y,z)}{2}{\left(\theta -\theta_{0}\right)}^{2} \end{array}$$

By construction, $\theta_{0}$ is still a solution to the equations of motion and all identities from Section 3 will hold identically when evaluated for $\theta_{0}$, with the exception of (30), which, as the only quantity in the perturbation equations, depends on second order derivatives of the Lagrangian with respect to θ. Thus, the effective mass changes to

(57) $$\begin{array}{ccc} {\left(m_{\mathrm{eff}}^{\prime }\right)}^{2} & = & m_{\mathrm{eff}}^{2}+\frac{a}{\sqrt{|\tilde{G}|}}. \end{array}$$

Therefore, by a suitable choice of a(θ,t,x,y,z), any $m_{\mathrm{eff}}^{\prime }$ can be reproduced without changing the analog metric.

Of course, Equation (55) has to remain an analog model when considering deviations around $\theta_{0}$, including the Gibbs relation (18), which is the most crucial for the analog gravity construction to work. This, however, follows directly from the theorem of implicit functions, if $\theta_{0}$ is not a degenerate point in the space of solutions.

## 5. Analog Entanglement Entropy

The entanglement entropy in general is defined for a quantum system divided into two subsystems *A* and *B*. For the density of states matrix ρ=|Ψ〉〈Ψ|, we define the reduced density matrix for the subsystem *A* by taking a partial trace over the subsystem *B*, i.e., $\rho_{A}={\mathrm{tr}}_{B}\;|\Psi \rangle \langle \Psi |$. Then, the entanglement entropy is defined as

(58) $$\begin{array}{c} S_{A}=-{\mathrm{tr}}_{A}\;\left(\rho_{A}\log\rho_{A}\right). \end{array}$$

The quantity $S_{A}$ is the entropy for an observer who can access information only from the subsystem *A* and can receive no information from *B*. The subsystem *B* is analogous to the interior of a BH horizon for an observer outside of the horizon. However, it is often not easy to compute the entanglement entropy, in particular in field theory in 3+1 or higher dimensions.

As discussed previously, the prescription for our analog model is only valid from the point $z_{\min}$ up to the horizon location at z=ℓ. Hence, we place the boundary of our model space–time at $z_{\min}$ and cut off the section from z=0 to $z_{\min}$ as it has been done for AdS5 in the Randall–Sundrum model [30,31]. The plane at $z=z_{\min}$ defines the boundary of our analog space–time, similar to the boundary of AdS space–time at z=0. Thus, our system is divided in two subsystems, *A* and *B*, where *A* extends from $z_{\min}$ up to the BH horizon at z=ℓ and *B* from z=0 to $z=z_{\min}$. Hence, the concept of entanglement entropy arises naturally in our analog model.

A convenient description of the entanglement entropy is derived in an *n*+1-dimensional field theory. It has been shown that the leading term of the entanglement entropy can be expressed as the area law [32,33]

(59) $$S_{A}=a\frac{\mathrm{Area}(\partial A)}{\ell^{n-1}}+\mathrm{subleading}\mathrm{terms},$$

where ∂A is the boundary of *A*, *ℓ* is an ultraviolet cutoff or the minimal length in the theory, and *a* is a constant which depends on the system. It is not accidental that this area law is of the same form as the Bekenstein–Hawking entropy of BHs in 3+1 dimensions, which is proportional to the area of the event horizon, with the constants n=3, a=1/4, and *ℓ* equal to the Planck length.

As we are dealing with an analog geometry, we will assume the existence of a minimal length. This length is typically of the order of the atomic separation. Below this scale, the bulk description of the fluid fails. This length describes the distance over which the wave function of a BE condensate tends to its bulk value when subjected to a localized perturbation. It is referred to as the healing length [34]. In analog gravity systems, a healing length $\ell_{\mathrm{hl}}$ plays the role of the Planck length [35,36,37,38,39] and, for a BE gas, is typically of order $\ell_{\mathrm{hl}}≃1/\left(mc\right)$, where *m* is the boson mass and *c* is the sound speed.

The entropy–area relation arises in the context of AdS/CFT duality. According to AdS/CFT, the entanglement entropy, being basically tied to the gravity in the bulk, should reflect fundamental features of the boundary gauge theory. In this regard, we will study the so-called ‘holographic entanglement entropy’ in 3+1 dimensions in the analog gravity context. In contrast to the usual entanglement entropy, for holographic entanglement entropy, the area of a fixed two-dimensional subsystem on the boundary depends on the geometry in the bulk. We expect that the holographic entanglement entropy in the analog model discussed in Section 4 should exhibit the features of the analog planar BH horizon.

The holographic entanglement entropy *S* in a 2+1-dimensional boundary field theory is defined for a two-dimensional subsystem Σ that has an arbitrary one-dimensional boundary ∂Σ. To calculate the entanglement entropy in our analog system, we use the area law prescription [40,41]

(60) $$S=\frac{\mathrm{Area}(\Sigma )}{4\ell^{2}}.$$

Here, Σ is the two-dimensional static minimal-area surface in the 3+1-dimensional bulk with boundary ∂Σ and we will identify the scale *ℓ* with $\ell_{\mathrm{hl}}$.

**Figure 1:**
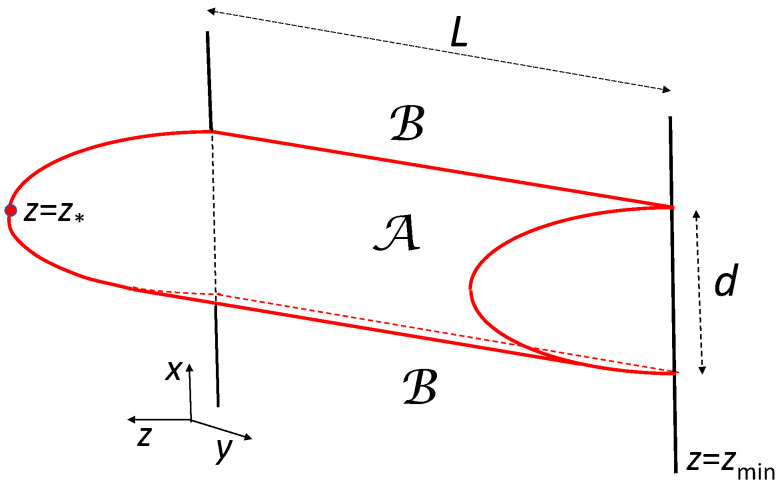
Strip geometry employed to calculate the entanglement entropy. Adapted illustration from Ref. [19].

We will apply the prescription from (60) to the strip geometry suggested in Ref. [40] (see also [21]), illustrated in Figure 1, and calculate the entropy *S* as a function of the strip width *d*.

Consider the bulk metric (Metric (4)) with n=3 and a surface Σ defined by the equation

(61) z−z(x)=0.

Here, z(x) is a function of *x* such that Σ extends into the bulk and is bounded by the perimeter of A as illustrated in Figure 1. The induced metric $\sigma_{ij}$ on Σ defines the line element

(62) $$ds_{\Sigma }^{2}=\sigma_{ij}dx^{i}dx^{j}=\frac{1}{\sqrt{1-\gamma (z)}}\left[dx^{2}\left(1+\frac{{z^{\prime }}^{2}}{\gamma (z)}\right)+y^{2}\right].$$

Finding the minimal area of Σ is equivalent to maximizing the functional

(63) $$I\left[z,z^{\prime }\right]=-\mathrm{Area}\left(\Sigma \right)/L=-\int dxdy\sqrt{\det\sigma_{ij}}/L=\int_{-d/2}^{d/2}dxL.$$

Here, *L* and *d* are respectively the length and width of the strip, and

(64) $$L=-\frac{1}{\sqrt{1-\gamma }}{\left(1+\frac{{z^{\prime }}^{2}}{\gamma }\right)}^{1/2}$$

The extremum condition δI=0 yields the equation of motion for *z*. We will employ the fact that the equation of motion is satisfied if and only if the Hamiltonian is a constant of motion. Using the conjugate momentum

(65) $$\pi =\frac{\partial L}{\partial z^{\prime }},$$

the Hamiltonian is defined as

(66) $$H=\pi z^{\prime }-L=\frac{1}{\sqrt{1-\gamma }}\frac{1}{{\left(1+{z^{\prime }}^{2}/\gamma \right)}^{1/2}}.$$

Since $z=z_{*}$ and $z^{\prime }=0$ at the bottom of the surface, we obtain the equation

(67) $$\frac{1}{\sqrt{1-\gamma (z_{*})}}=\frac{1}{\sqrt{1-\gamma (z)}}\frac{1}{{\left(1+{z^{\prime }}^{2}/\gamma \left(z\right)\right)}^{1/2}},$$

from which we can express $z^{\prime }$ as

(68) $$z^{\prime }=\pm \frac{\sqrt{\gamma \left(z\right)(\gamma \left(z\right)-\gamma \left(z_{*}\right))}}{\sqrt{1-\gamma (z)}}.$$

Inserting this into (63) and changing the integration variable from *x* to *z* with $dx=dz/z^{\prime }$, we obtain the entanglement entropy expressed as an integral over *z*:

(69) $$S=\frac{\mathrm{Area}}{4\ell^{2}}=\frac{L}{2\ell^{2}}\int_{z_{\min}}^{z_{*}}dz\frac{\sqrt{1-\gamma (z_{*})}}{\sqrt{1-\gamma (z)}}\frac{1}{\sqrt{\gamma \left(z\right)(\gamma \left(z\right)-\gamma \left(z_{*}\right))}}.$$

The location of the bottom $z_{*}$ of the extremal surface is related to the strip width

(70) $$d=2\int_{-d/2}^{d/2}dx=2\int_{z_{\min}}^{z_{*}}dz\frac{\sqrt{1-\gamma (z)}}{\sqrt{\gamma \left(z\right)(\gamma \left(z\right)-\gamma \left(z_{*}\right))}}.$$

Given the blackening metric function γ, Equations (69) and (70) define the entropy *S* as a parametric function of the strip width *d* with the parameter $z_{*}$ ranging from $z_{\min}$ to *ℓ*.

By way of example, we numerically compute the function S=S(d) for a planar AdS_5_ BH with γ as in Equation (32). Since the analog metric is 3+1-dimensional, we will ignore the fifth space coordinate, so the boundary at $z=z_{\min}$ will be a two-dimensional space-like plane. In the limit $z_{*}\to \ell$ both *S* and *d* diverge logarithmically. It may be easily shown that in this limit the function S=S(d) asymptotically approaches the linear function

(71) $$S_{\lim}=\frac{L}{2\ell }\left(3^{1/4}-1+\frac{d}{2\ell }\right).$$

**Figure 2:**
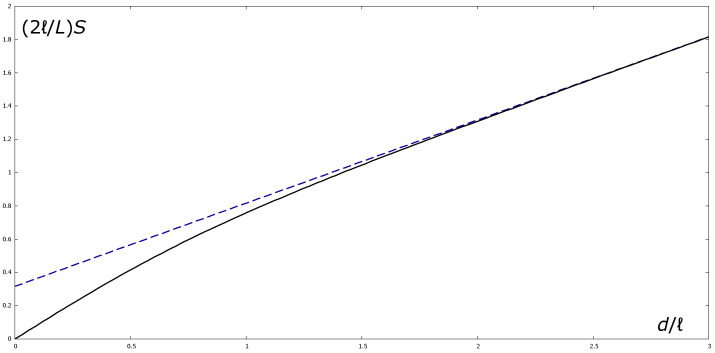
Holographic entanglement entropy (full black line) and limiting function $S_{\lim}$ (blue dashed line) versus strip width.

Hence in the limit of large *d*, the entanglement entropy obeys the area law in (59) with a=1/4 and a subleading term equal to $\left(3^{1/4}-1\right)L/\left(2\ell \right)$. In Figure 2, we plot both functions S(d) and $S_{\lim}\left(d\right))$ in units of L/(2ℓ).

## 6. Summary and Conclusions

Using the formalism of analog gravity for the case of nonisentropic fluids from [10], we have shown that by a suitable transformation of variables and choice of parametrization, a Lagrangian of the form in (6) is an analog model for a scalar field propagating in a space–time that is conformal to a static, planar BH space–time. We have also demonstrated how, with a suitable adjustment of the external potential that couples to the analog Lagrangian, it is possible, for any given analog metric, to simulate an arbitrary effective mass for the perturbation. Furthermore, we have studied the analog entanglement entropy and computed it numerically for an analog planar AdS_5_ BH. These results are valid for a generic choice of conformal rescaling and blackening factor of the metric, for an arbitrary effective mass of the scalar perturbation.

It is worth noting that our acoustic metric is specified completely by three independent functions: z-component of the velocity, the density ρ, and the pressure *p* specified by the equation of state p=p(ρ). Furthermore, the equation of continuity reduces these three degrees of freedom to two. Hence, as in a general acoustic geometry (see, e.g., Ref. [2]), our analog geometry has two degrees of freedom per point in space–time in contrast to the 3+1-dimensional pseudo-Riemannian geometry where the metric has six degrees of freedom.

The procedure outlined here allows for vastly extending the class of phenomena in gravity physics that can be simulated in condensed matter systems via the analog gravity formalism. Additionally, the effects we have discussed may be of phenomenological interest in all those phenomena that involve relativistic fluids under extreme conditions. For example, this may be the case in ultrarelativistic heavy-ion collisions, where the fluid of particles is predominantly produced along one space dimension.

This class of phenomena has now been shown to include most non-rotating planar BH metrics considered in the literature—as well as several cosmological space–times of particular interest. As our emphasis was put on planar BH geometries, our result also provides new foundations for the surge of investigations on how analog gravity interlinks with gauge/gravity duality (for reviews see [16,42]) and condensed matter physics in recent years [29,43,44,45,46,47], where the type of space–times considered here also plays a central role.

We emphasize again that the main difference between our analog model and analog planar models considered in the literature, e.g., in Refs. [10,11,12,13], is in our study of a generic stationary planar BH metric. Besides, we provide a prescription for calculating the holographic entanglement entropy for a general analog planar BH space–time with an AdS asymptotic boundary. A generalization to geometries with spherical or axial symmetry is possible and relatively straightforward, but will be left for future work.

## Data Availability

The original contributions presented in this study are included in the article. Further inquiries can be directed to the corresponding author.
